# Extensive aortic thrombosis and testicular infarction – a rare complication of biventricular cardiac thrombi

**DOI:** 10.1093/omcr/omac073

**Published:** 2022-07-26

**Authors:** Saad M Ezad, Andrew Salmon, Hooria Cheema, Rosie Swallow

**Affiliations:** Cardiovascular Division, King’s College London, London, UK; Dorset Heart Centre, Royal Bournemouth Hospital, Bournemouth, UK; Dorset Heart Centre, Royal Bournemouth Hospital, Bournemouth, UK; Radiology Department, University Hospital Southampton, Southampton, UK; Dorset Heart Centre, Royal Bournemouth Hospital, Bournemouth, UK

## Abstract

Heart failure is a heterogenous syndrome which is increasing in prevalence, with a prognosis worse than many malignancies. Morbidity and mortality most commonly occur secondary to pump failure or ventricular arrhythmias; however, a more infrequently seen complication is the formation of mural thrombi. More commonly seen within the left ventricle, thrombi can embolize leading to stroke or end organ infarction. We present the case of a male who presented with decompensated heart failure. The presence of biventricular thrombi was found on echocardiography and subsequent cross-sectional imaging revealed these had embolized resulting in the rare complication of extensive abdominal aortic thrombosis with renal and testicular infarction. Biventricular thrombi are rare but high risk due to the potential for embolization as demonstrated in this case. Prompt recognition and management with anti-coagulation are essential, followed by treatment of the underlying pathology, which resulted in the formation of thrombi to prevent recurrence.

## INTRODUCTION

Heart failure is an epidemic resulting in significant health care expenditure and morbidity for patients. Mural thrombus is a rare complication most commonly seen in the left ventricle (LV) following anterior myocardial infarction. Biventricular thrombi have been infrequently described; we present a case of biventricular thrombi resulting in extensive embolization and end organ infarction.

## CASE REPORT

A 57-year-old male smoker presented with exertional dyspnoea and scrotal swelling, without testicular pain, on a background of hypertension and excessive alcohol consumption. Physical examination was consistent with decompensated heart failure, including elevated jugular venous pressure, anasarca, peripheral and scrotal oedema.

**Figure 1:**
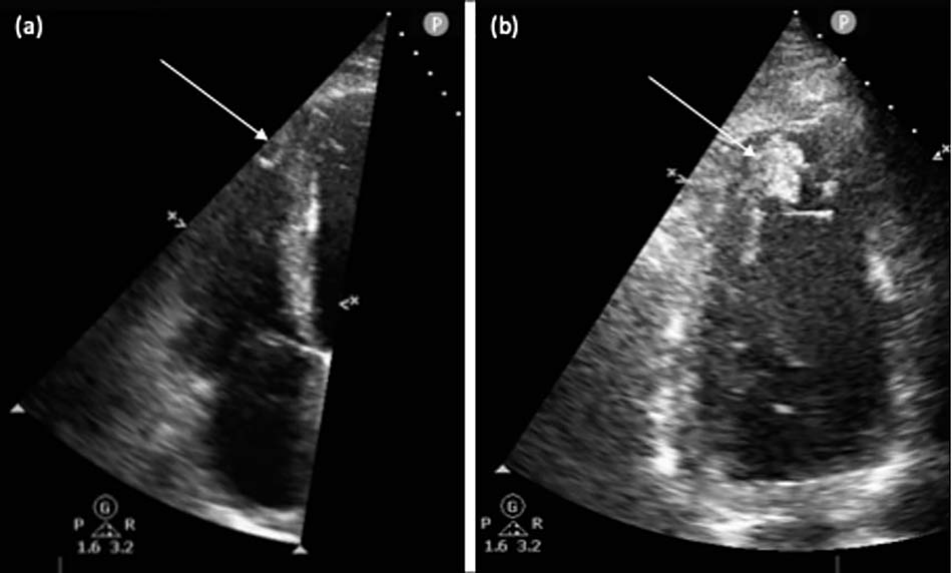
Transthoracic echocardiogram. (**a**) Focused RV view with thrombus visualized in the apex (arrow). (**b**) Two-chamber view of the LV with thrombus visualized in the apex (arrow).

Initial blood tests revealed an N-terminal pro B-type natriuretic peptide level of 27 594 pg/ml (<400 pg/ml) with deranged liver and renal biochemistry including an alanine aminotransferase (ALT) of 2266 u/l (<50 u/l) and an estimated glomerular filtration rate of 35 ml/min (>90 ml/min). Echocardiogram confirmed LV systolic impairment with an ejection fraction of 10%, moderately dilated LV and right ventricular (RV) cavities and severe functional mitral regurgitation. In addition, apical thromboses were present in the LV and RV (Fig. 1, Video 1 and Video 2).

**Figure 2:**
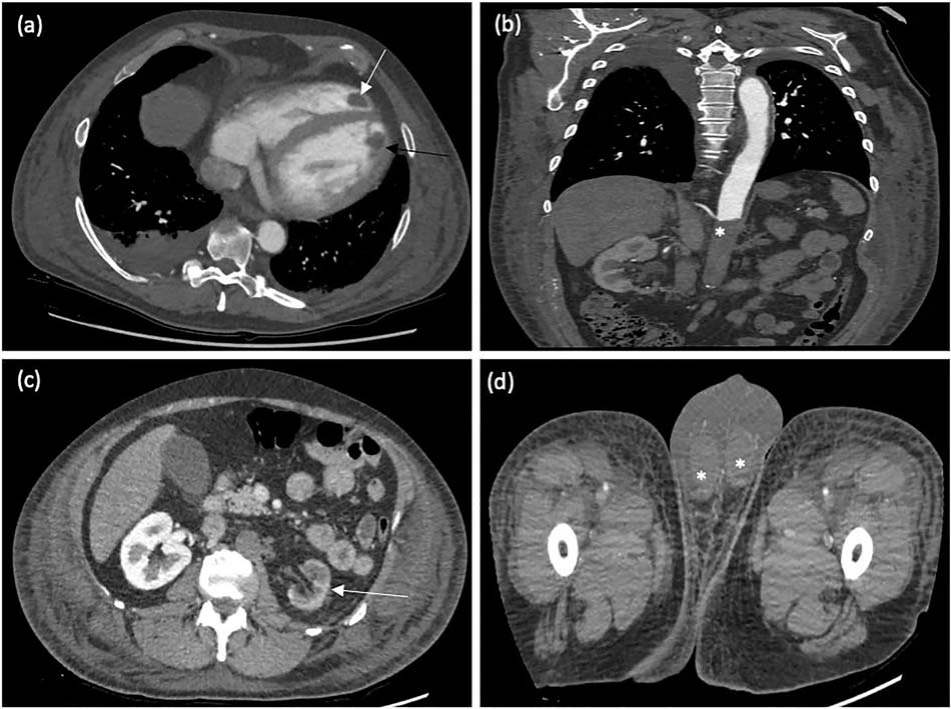
(**a**) Axial contrast-enhanced CT chest demonstrating biventricular apical thrombi (white arrow—RV thrombus, black arrow—left ventricular thrombus). (**b**) Coronal contrast-enhanced CT abdomen demonstrating extensive occlusive thrombus within the abdominal aorta (^*^). (**c**) Axial contrast-enhanced CT abdomen demonstrating reduced enhancement of the left kidney in keeping with infarction (Arrow). (**d**) Axial contrast-enhanced CT abdomen demonstrating reduced enhancement of the testes bilaterally in keeping with infarction (^*^).

Given the presentation of gross peripheral oedema, scrotal oedema and deranged ALT, a post-contrast CT chest, abdomen and pelvis was performed to exclude metastatic disease, specifically liver metastases. The CT study confirmed biventricular dilatation and apical thromboses (Fig. 2**a**). No evidence of malignancy or metastatic disease was demonstrated; however, important incidental findings included extensive occlusive abdominal aortic thrombus, which extended from the level of the superior mesenteric artery into the distal external iliac arteries where there was re-opacification of the vessels (Fig. 2**b**).

The left renal artery was completely occluded with reduced enhancement of the left kidney, consistent with infarction (Fig. 2**c**). The inferior mesenteric artery was also occluded at its origin although there was distal opacification and normal mucosal enhancement of the colon was preserved. In addition, there was reduced enhancement of the testes bilaterally with occlusion of the testicular arteries (Fig. 2**d**).

Given the history of excessive alcohol consumption, this was presumed to be the most likely aetiology of the patient’s heart failure which subsequently resulted in the formation of biventricular thrombi leading to embolic extensive aortic thrombosis. Ischaemic cardiomyopathy was deemed unlikely given the absence of anginal symptoms or ischaemic electrocardiogram changes. Idiopathic dilated cardiomyopathy would be an alternative differential diagnosis for this patient’s presentation. An underlying thrombophilia such as anti-phospholipid syndrome accounting for the multiple sites of arterial thrombosis was considered; however, a thrombophilia screen was negative.

A multifaceted management approach to this patient initially included treatment of heart failure with intravenous furosemide infusion and commencement of ramipril. His heart failure medications were further optimized with the addition of eplerenone and ivabradine with good symptomatic benefit. The patient was strongly encouraged to cease further alcohol consumption.

With regards to the extensive aortic thrombosis, a surgical approach was deemed prohibitively high risk given the patient’s poor cardiac function. A percutaneous thrombectomy via the femoral approach was attempted but was unsuccessful, and therefore the patient was managed conservatively with a heparin infusion and subsequently converted to warfarin.

Serum testosterone levels were found to be low at 1.3 nmol/L (7–26 nmol/L) due to testicular infarction, and therefore replacement was commenced. Following improvement in symptoms and renal and liver biochemistry, the patient was discharged on oral diuretics and long-term warfarin therapy with an INR target of 2–3 in keeping with guideline recommendations [1].

Follow-up.

The patient developed sepsis $\sim$ 2 months following the initial presentation, from the right femoral access site which was persistently discharging following the previous attempt at femoral thrombectomy. A post-contrast CT chest, abdomen and pelvis study was performed to exclude other potential sources of sepsis, confirmed a right groin collection. There was persistent extensive aortic occlusive thrombus as seen previously; however, there was evidence of new collateralization from rectus abdominis and paraspinal arteries filling the external iliac and femoral arteries. The biventricular thrombi had resolved and there was no evidence of propagation of the aortic thrombus or new arterial thrombosis.

## DISCUSSION

The formation of LV thrombus in the context of cardiomyopathy with LV impairment and reduced ejection fraction is well-documented; however, biventricular thrombosis is a rarer occurrence complicating up to 23.9% of LV thrombi [2]. Biventricular thrombi have been previously reported in the literature complicating acute myocardial infarction, cardiomyopathy, myocarditis and pulmonary embolism [2–4]. The proposed mechanism for ventricular thrombus formation in the context of dilated cardiomyopathy, as in this case, is blood stasis secondary to cavity dilatation and hypokinesis. A hypercoagulable state can also predispose to the formation of biventricular thrombi including conditions such as antiphospholipid syndrome and heparin-induced thrombocytopaenia. A thrombophilia screen should therefore be performed as part of the investigation for biventricular thrombi.

Complications of biventricular thrombi include systemic and pulmonary embolization which can result in significant end-organ damage such as pulmonary emboli, stroke, acute limb ischaemia and visceral ischaemia. Contemporary data suggest an annualized embolism rate of 3.7% despite treatment with anti-coagulation [5]. Ventricular thrombi complicated by such extensive aortic thrombus have not previously been reported in the literature to our knowledge. There was evidence of end-organ damage with left renal infarct and bilateral testicular infarcts. No pulmonary emboli were demonstrated in this case.

A high index of suspicion is required when predisposing factors for the formation of ventricular thrombi exist such as apical hypokinesia particularly following a large anterior myocardial infarction. Transthoracic echocardiography (TTE) is the initial investigation of choice and has 35–60% sensitivity and 90% specificity for LV thrombus detection. Although CT has similar detection rates to TTE, delayed enhancement cardiac MRI is the gold standard for LV thrombus detection with 88% sensitivity and 99% specificity [6].

There are no guideline recommendations for the treatment of biventricular thrombus. In practice, patients are treated with low molecular weight heparin before being established on oral anticoagulation. Warfarin is the only approved oral anticoagulant for LV thrombus; however, emerging evidence suggests direct oral anticoagulants may have a role in selected patients [7]. Current European Society of Cardiology guidelines recommend oral anticoagulation for up to 6 months for treatment of LV thrombus, with duration guided by surveillance TTE and consideration of bleeding risk [8]. Thrombolysis remains a treatment option, however carries a risk of catastrophic embolization [3]. Thrombectomy should be considered in patients with a larger, more mobile thrombi which carry an increased risk of embolization [9].

Conservative management in the present case with warfarin resulted in the resolution of ventricular thrombi; however, due to persisting aortic thrombus, the decision was made to continue long-term anti-coagulation.

## CONFLICT OF INTEREST STATEMENT

No conflicts of interest.

## FUNDING

There were no sources of funding associated with this case report.

## ETHICAL APPROVAL

No ethical approval was required for this case report.

## CONSENT

Written patient consent was obtained for the publication of this case report.

## GUARANTOR

Dr Saad Ezad.

## Supplementary Material

Video_1_omac073

Video_2_omac073
